# Extracellular vesicles from deciduous pulp stem cells recover bone loss by regulating telomerase activity in an osteoporosis mouse model

**DOI:** 10.1186/s13287-020-01818-0

**Published:** 2020-07-17

**Authors:** Soichiro Sonoda, Sara Murata, Kento Nishida, Hiroki Kato, Norihisa Uehara, Yukari N. Kyumoto, Haruyoshi Yamaza, Ichiro Takahashi, Toshio Kukita, Takayoshi Yamaza

**Affiliations:** 1 Department of Molecular Cell Biology and Oral Anatomy, Division of Oral Biological Sciences, Graduate School of Dental Science, Kyushu University, 3-1-1 Maidashi, Higashi-ku, Fukuoka, 812-8582 Japan; 2 Section of Orthodontics and Dentofacial Orthopedics, Division of Oral Health, Growth & Development, Faculty of Dental Science, Kyushu University, Fukuoka, Japan; 3 Department of Pediatric Dentistry, Division of Oral Health, Growth & Development, Graduate School of Dental Science, Kyushu University, Fukuoka, Japan

**Keywords:** Human deciduous pulp stem cells, Extracellular vesicles, MicroRNA, Telomerase activity, Osteoporosis

## Abstract

**Background:**

Systemic transplantation of stem cells from human exfoliated deciduous teeth (SHED) recovers bone loss in animal models of osteoporosis; however, the mechanisms underlying this remain unclear. Here, we hypothesized that trophic factors within SHED-releasing extracellular vesicles (SHED-EVs) rescue osteoporotic phenotype.

**Methods:**

EVs were isolated from culture supernatant of SHED. SHED-EVs were treated with or without ribonuclease and systemically administrated into ovariectomized mice, followed by the function of recipient bone marrow mesenchymal stem cells (BMMSCs) including telomerase activity, osteoblast differentiation, and sepmaphorine-3A (SEMA3A) secretion. Subsequently, human BMMSCs were stimulated by SHED-EVs with or without ribonuclease treatment, and then human BMMSCs were examined regarding the function of telomerase activity, osteoblast differentiation, and SEMA3A secretion. Furthermore, SHED-EV-treated human BMMSCs were subcutaneously transplanted into the dorsal skin of immunocompromised mice with hydroxyapatite tricalcium phosphate (HA/TCP) careers and analyzed the de novo bone-forming ability.

**Results:**

We revealed that systemic SHED-EV-infusion recovered bone volume in ovariectomized mice and improved the function of recipient BMMSCs by rescuing the mRNA levels of *Tert* and telomerase activity, osteoblast differentiation, and SEMA3A secretion. Ribonuclease treatment depleted RNAs, including microRNAs, within SHED-EVs, and these RNA-depleted SHED-EVs attenuated SHED-EV-rescued function of recipient BMMSCs in the ovariectomized mice. These findings were supported by in vitro assays using human BMMSCs incubated with SHED-EVs.

**Conclusion:**

Collectively, our findings suggest that SHED-secreted RNAs, such as microRNAs, play a crucial role in treating postmenopausal osteoporosis by targeting the telomerase activity of recipient BMMSCs.

## Background

Stem cells from human exfoliated deciduous teeth (SHED) are isolated from the pulp tissues of deciduous teeth and exhibit self-renewal properties and differentiate into osteoblasts, chondrocytes, adipocytes, neural cells, endothelial cells, and hepatocytes [1, 2]. SHED also exhibit improved properties including colony formation, proliferation, and immunomodulatory function and are less tumorigenic than bone marrow mesenchymal stem cells (BMMSCs) [3, 4]. Human clinical trials aimed at treating trauma-induced tooth injury [5] as well as animal pre-clinical studies for diseases including systemic lupus erythematosus (SLE), spinal injury, and Wilson’s disease [2, 3, 6, 7] have reported that SHED are promising candidates for cell-based therapies [8]. Systemic SHED transplantation-based therapy employs multiple mechanisms, including direct conversion into target tissue-specific cells, release of trophic factors, and cell-cell contact [3, 6, 7, 9–11].

Recent studies have also shown that systemic SHED transplantation increases bone volume in SLE-model *Fas*-mutated MRL/*lp*r mice and postmenopausal osteoporosis model ovariectomy-induced (OVX) mice, but the frequency of engrafted SHED in recipient bone tissues was very low [12, 13]. Further, the interplay between SHED and other immune cells, especially T cells, via the Fas ligand-Fas pathway improves osteoporotic phenotype in OVX mice [13]. Osteoporotic condition in MRL/*lp*r and OVX mice impairs the functions of endogenous BMMSCs [14, 15]. Specifically, systemic SHED transplantation rescues recipient BMMSC function in MRL/*lp*r and OVX mice [12–15]. However, the mechanism of rescuing recipient BMMSC function underlying SHED transplantation remains unclear.

Telomerase reverse transcriptase (TERT) is the main catalytic telomerase subunit that is required to maintain telomere length [16]. Although telomerase activity is undetectable in most normal human somatic cells, it acts at varying degrees in stem/progenitor cells [17], which induces feedback regulation [18]. This activity confers properties of self-renewal, proliferation, osteogenic differentiation, and tissue regeneration in mesenchymal stem cells (MSCs) [19–21] and is also involved in immunomodulating donor MSCs to improve systemic sclerosis-like symptoms in Tsk/^+^ mice [22]. However, it is not fully understood whether telomerase activity of recipient organs/tissues/cells can be targeted in SHED-based therapy.

Extracellular vesicles (EVs) are membrane-bound vesicles secreted from cells that exist as either exosomes (40–100 nm in diameter) or plasma membrane-derived microvesicles (100–1000 nm in diameter). Stem cell-derived EVs contain enriched small RNAs, mostly microRNAs (miRNAs) as compared to parental cells [23], and participate in intercellular communication and therapeutic effects [24]. SHED are able to release EVs including exosomes and microvesicles [25]. Administration of SHED-EVs abrogates disease-specific disorders associated with acute inflammation, brain injury, and Parkinson’s disease [26–28]. However, the therapeutic efficacy and mechanisms of SHED-EV-based cell-free therapy remain unclear in postmenopausal osteoporosis. Thus, in the present study, we identified important roles of SHED-EVs in rescuing the recipient BMMSC function via activation of telomerase activity, and we suggest that SHED-EV-based cell-free therapy might be a novel therapeutic strategy for postmenopausal osteoporosis.

## Methods

### Mice

C57BL/6J mice (female, 8 weeks old) and Balb/c nu/nu mice (female, 10 weeks old) were purchased from CLEA Japan, Inc. (Tokyo, Japan). All animal experiments were approved by the Institutional Animal Care and Use Committee of Kyushu University (Approval Number: A21-044-1).

### Antibodies

Additional file 1: Supplementary Table 1 lists the antibodies used in this study.

### Isolation, culture, and characterization of SHED, human BMMSCs, and mouse BMMSCs

SHED, human BMMSCs (hBMMSCs), and mouse BMMSCs (mBMMSCs) were isolated and cultured according to previous reports [1, 29]. The isolated cells were characterized as MSCs according to the published criteria [30]. The details have been described in Additional file 1: Supplementary Methods.

### Isolation, characterization, and ribonuclease A treatment of SHED-EVs

Conditioned medium (CM) was collected from 3-day SHED cultures with Dulbecco’s minimum essential medium low glucose type (Thermo Fisher Scientific, Waltham, MA). The CM was centrifuged at 500×*g* for 5 min and used for SHED-EV isolation using the exoEasy Maxi kit (Qiagen, Valencia, CA) according to the manufacturer’s protocol. SHED-EV particle size was measured using the qNano analyzer (Izon Science, Christchurch, New Zealand). A fraction of the SHED-EVs were treated with ribonuclease A (RNase A; 5 U/mL; Thermo Fisher Scientific) at 37 °C for 3 h and incubated with RNase inhibitor (40 U/mL; Thermo Fisher Scientific) at room temperature for 10 min followed by ultracentrifugation at 110,000×*g* for 1 h. SHED-EVs were subjected to flow cytometry (FCM) analysis using the ExoAB Antibody kit (ExoAB-KIT-1, System Bioscience, Palo Alto, CA) and R-phycoerythrin-conjugated anti-rabbit IgG (Cell Signaling Technology, Danvers, MA) according to the manufacturer instructions. Total proteins were extracted from the SHED-EVs and SHED using the M-PER mammalian protein extraction reagent (Thermo Fisher Scientific) with proteinase inhibitor cocktail (Nacalai Tesque) and quantified using a Bio-Rad protein assay (Bio-Rad, Hercules, CA) following which they were used for Western blotting. Total RNA was extracted from SHED-EVs using a miRNeasy Mini kit (Qiagen) according to the manufacturer’s instructions. RNA quality and quantity were determined using the Agilent 2100 Bioanalyzer (Agilent Santa Clara, CA).

### Systemic infusion of SHED-EVs into mice with postnatal osteoporosis

Ovariectomized female C57BL/6J mice (10 weeks old; OVX mice) were intravenously administered SHED-EVs (100 μg in 100 μL PBS) pretreated with or without ribonuclease A (RNase) 2 days post-surgery and sacrificed 4 weeks post-surgery. Age-matched sham-operated C57BL/6J and OVX mice infused with PBS (100 μL/10 g body weight) served as experimental controls.

### In vivo and in vitro tracing assays

Carboxyfluorescein diacetate succinimidyl ester (CFSE; Thermo Fisher Scientific) or PBS was used for labeling according to the kit instructions. CSFE-labeled SHED-EVs (100 μg in 100 μL PBS) were intravenously infused into OVX mice (10 weeks old) 2 days post-surgery. After 3 days of infusion, prepared frozen sections were mounted using Vectashield mounting medium containing 4′,6-diamidino-2-phenylindole (Vector Laboratories, Burlingame, CA, USA). CSFE-labeled SHED-EVs (20 μg/mL) were incubated with cultured hBMMSCs for 3 days and were subjected to histological and FCM analyses.

### Bone analysis by micro-computed tomography

We used third vertebral bodies for bone assays, because of the difference of strain and skeletal site on bone reduction and therapeutic action in mouse estrogen-deficient condition [31, 32]. Bone mineral density (BMD) and bone structural indices including trabecular bone volume versus total volume (BV/TV), trabecular numbers (Tb.N), and trabecular thickness (Tb.Th) of mouse third lumbar vertebrae (L3) were analyzed using a 1076 micro-computed tomography (micro-CT) system micro-CT scanner (Skyscan, Kontich, Belgium) and the CTAn software (Skyscan) as described previously [12]. Density values were calibrated using hydroxyl apatite phantoms with BMD values of 0.25 and 0.75 g/cm^3^ (Skyscan).

### Telomerase activity analysis

Telomerase activity was analyzed by quantitative telomerase repeated amplification protocol (RQ-TRAP) using a quantitative telomerase detection kit (Allied Biotech, Inc., Ijamsville, MD) according to the manufacturer’s instructions as reported previously [29]. HEK293T cells (Thermo Fisher Scientific) were used as the positive control. Heat-inactivated cell lysates were used as negative controls.

### Colony forming units-fibroblast assays

Colony forming units-fibroblast (CFU-F) assays was performed as described in Additional file 1: Supplementary Methods.

### Cell proliferation assay

Bromodeoxyuridine (BrdU)-uptake assay was performed as described in Additional file 1: Supplementary Methods.

### Surface antigen analysis

BMMSCs (0.1 × 10^6^) were stained with R-phycoerythrin-conjugated antibodies (1 μg) or isotype-matched control antibodies and analyzed by FCM as described in Additional file 1: Supplementary Methods.

### In vitro osteoblast function of mouse and human BMMSCs

Mouse and human BMMSCs were cultured under osteogenic condition [29]. Gene and protein expression of osteoblast functional markers were assayed by reverse transcription-quantitative polymerase chain reaction (RT-qPCR), Western blot analyses, and enzyme-linked immunosorbent assay (ELISA) 1 week after the osteogenic induction. Mineralized tissue formation was analyzed by Alizarin Red-S staining 4 weeks after the osteogenic induction. Seven representative images were randomly selected to measure the area of Alizarin Red-S-positive area per total culture disk area using the ImageJ software (National Institutes of Health, Bethesda, MA).

### RT-qPCR

RT-qPCR was performed as described in Additional file 1: Supplementary Methods.

### Western blot analysis

Western blot analysis was performed as described in Additional file 1: Supplementary Methods.

### ELISA

ELISA was performed as described in Additional file 1: Supplementary Methods.

### Statistical analyses

The data were expressed as mean ± standard error of the mean or mean ± standard deviation from triplicates. Comparisons between two groups were analyzed by an independent two-tailed Student’s *t* test. Multiple group comparisons were performed by a one-way analysis of variance followed by Tukey’s post hoc test. Kaplan-Meier and Kruskal-Wallis tests were used for the survival assays. *P* < 0.05 were considered statistically significant. All statistical analyses were performed using the PRISM 6 software (GraphPad, Software, La Jolla, CA, USA).

## Results

### Systemic SHED transplantation rescues impaired BMMSC function through telomerase activity in recipient OVX mice

In this study, female C57BL/6 mice (10 weeks old) were ovariectomized and used to understand whether estrogen deficiency affects BMMSCs. To understand the stemness of BMMSCs, we analyzed the capacities of colony formation, cell proliferation, and cell surface antigen expression. CFU-F and BrdU assays showed enhanced colony formation and decreased proliferation, respectively, in BMMSCs derived from OVX mice (OVX-BMMSCs) in comparison to BMMSCs derived from Sham-mice (Sham-BMMSCs) (Additional file 1: Supplementary Figure 1a). CD146 have been reported as a strong candidate for a critical marker of MSCs because of the self-renewing and tissue organizing ability in osteogenic progenitors [33]. Therefore, we examined the expression of the primitive marker CD146 in recipient BMMSCs for evaluating their stemness and function. FCM analysis revealed that only CD146 and CD73 levels were decreased in OVX-BMMSCs compared to Sham-BMMSCs (Additional file 1: Supplementary Figure 1b). To assess the function of BMSSCs, we analyzed osteoblast function. Alizarin Red staining and RT-qPCR revealed that OVX-BMMSCs exhibited impaired function in osteogenic inductive conditions as observed by a reduction in the formation of mineralized nodules and mRNA levels for osteoblast markers, including Runt-related transcription factor 2 (*Runx2*) and bone gamma carboxyglutamic acid protein (*Bglap*), and osteoprotective factor semaphorin-3a (*Sema3a*) (Additional file 1: Supplementary Figures 1c, 1d). These findings indicated that OVX-BMMSCs were impaired in an estrogen-deficient condition.

Hence, in this study, OVX mice received SHED (0.1 × 10^6^/10 g body weight) 2 days post-surgery and were used to determine the effects of estrogen deficiency on the recipient BMMSCs (SHED-transplanted mice-derived BMMSCs: SHED-BMMSCs) 4 weeks post-transplantation. Human MSCs are known to be highly heterogeneous, especially between different donors. Therefore, we tested three donor-derived SHED in ovariectomized mice. Meanwhile, CFU-F, BrdU, and FCM assays showed that SHED transplantation rescued the function of recipient SHED-BMMSCs as seen by decreased colony formation as well as increased cell proliferation, and CD146 and CD73 levels. We also detected increased mineralized nodule formation and mRNA levels of *Runx2*, *Bglap*, and *Sema3a* under osteogenic conditions, indicating recovery of the impaired function of recipient SHED-BMMSCs (Additional file 1: Supplementary Figures 1a–1d). Thus, SHED transplantation appears to rescue the impaired function of recipient BMMSCs in OVX mice.

Since low telomerase activity has been shown to be critical to regulate osteogenesis by BMMSCs [19, 21], we next examined the difference in telomerase activity between OVX-BMMSCs and Sham-BMMSCs. OVX-BMMSCs showed a significant decrease in telomerase activity in comparison with Sham-BMMSCs as determined by telomerase activity assay (Additional file 1: Supplementary Figure 2a). Interestingly, the telomerase activity of SHED-BMMSCs was recovered (Additional file 1: Supplementary Figure 2a). Additionally, TERT has been shown to play an essential role in regulating telomerase activity [16]. We, therefore, examined whether TERT contributes to the telomerase activity in recipient BMMSCs, and confirmed that SHED transplantation rescued the decreased *Tert* mRNA level in OVX-BMMSCs by RT-qPCR analysis (Additional file 1: Supplementary Figure 2b). Hence, these results indicate that the status of TERT-associated telomerase activity is crucial for the SHED transplantation-mediated rescue of impaired recipient BMMSC functioning in OVX mice.

### Systemic SHED transplantation recovers bone loss in postmenopausal OVX mice

Postmenopausal osteoporosis is a common systemic skeletal disease in elderly women that results from an imbalance between bone resorption by osteoclasts and bone formation by osteoblasts, thereby leading to a high risk factor for fragility fractures associated with reduced BMD and deteriorated bone microarchitecture [34]. Systemic SHED transplantation has been found to ameliorate bone reduction in an OVX mouse model for postmenopausal osteoporosis [12, 15]. Hence, in this study, SHED-transplanted OVX mice were used to determine the osteoporotic phenotype of the L3 4 weeks post-transplantation. Increased BMD, BV/TV, Tb. N, and Tb. Th were observed by micro-CT, indicating rescue of the osteoporotic phenotype in the treated OVX mice as compared to the control mice (Additional file 1: Supplementary Figure 3).

Enhanced in vivo osteoclast activity in OVX mice was rescued 4 weeks after SHED transplantation, as observed by the decrease osteoclast number as well as serum levels of the sRANKL and CTX following TRAP staining and ELISA (Additional file 1: Supplementary Figure 4). Further, the in vitro osteoclast activity of OVX mice was rescued 4 weeks after SHED transplantation, as indicated by the decrease in TRAP-positive multinuclear cells (MNCs) and mRNA levels of the osteoclast markers, including TNF receptor superfamily member 11a (*Tnfrsf11a*), nuclear factor of activated T cell (*Nfatc1*), and cathepsin K (*Ctsk*), by TRAP staining and RT-qPCR following co-culturing of calvarial osteoblasts from wild-type newborn C57BL/6 mice stimulated with prostaglandin E2 (Additional file 1: Supplementary Figure 5). These results suggest that systemic SHED infusion rescues osteoporotic phenotypes and improves the osteoclast-mediated bone loss.

### Infusion of SHED-secreted EVs ameliorates bone reduction in OVX mice

Although SHED were detected in the bone marrow 7 days after the infusion, CFSE labeling did not show a significant frequency of engrafted SHED in the recipient bone and bone marrow of OVX mice (Additional file 1: Supplementary Figure 6). Since SHED transplantation increased *Tert* mRNA levels to rescue telomerase activity in recipient BMMSCs, we hypothesized that SHED indirectly contribute to the SHED transplantation-mediated rescue of impaired *Tert* mRNA expression, telomerase activity, and bone loss in OVX mice. Particularly, trophic factors within MSC-secreting EVs mediate cell-cell communication to deliver extracellular signals, thereby leading to therapeutic effects [35, 36].

**Fig. 1:**
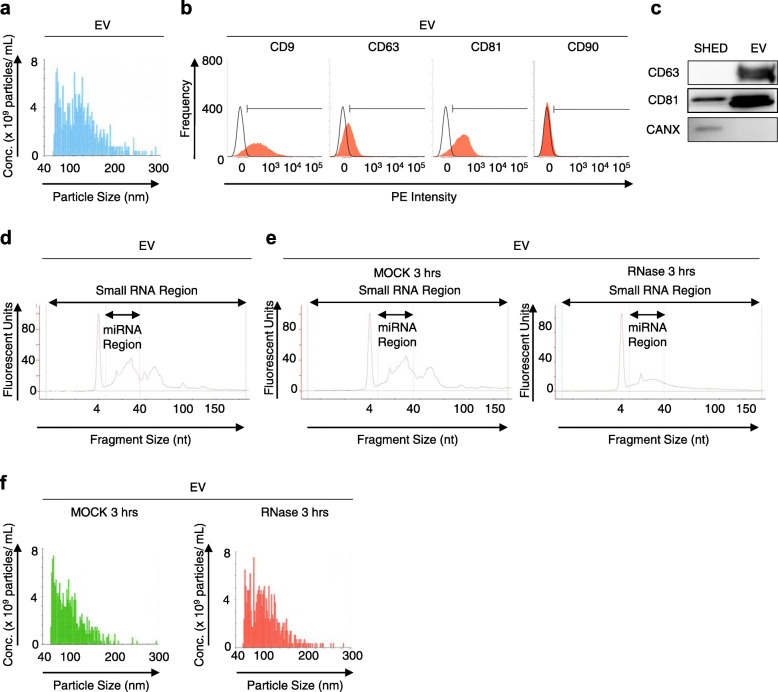
Characterization of SHED-derived extracellular vesicles (SHED-EVs). SHED-EVs were isolated from SHED-conditioned medium harvested from 3-day cultures. **a** Representative histogram of the particle size of SHED-EVs by nanotracking particle analysis. **b** Representative histograms of the expression of CD9, CD63, CD81, and CD90 in SHED-EVs by flow cytometric (FCM) analysis. PE, R-phycoerythrin. Areas filed with red, target antibody-stained histograms; solid lines, isotype-matched control-stained histograms. **c** Representative Western blotting images of the expression of CD63, CD81, and calnexin (CANX) in SHED and SHED-EVs. **d** Representative histograms of the small RNA and microRNA (miRNA) content within SHED-EVs. **e**, **f** SHED-EVs were treated with RNase A (5 U/mL; RNase) or PBS (MOCK) for 30 min. Representative histograms of the small RNA and miRNA content in SHED-EVs (**e**) and particle concentration per particle size of SHED-EVs (**f**)

We isolated EVs from the conditioned medium from three donor-derived SHED, which were the same three donors evaluated the therapeutic potential for ovariectomy-induced osteoporosis in mice (Additional file 1: Supplementary Figures 1–5), harvested from a 3-day culture using a commercial-available kit according to the manufacturer’s protocol, as reported previously [37]. To characterize the EVs from the conditioned medium of SHED (SHED-EVs), we examined the phenotype of them by multiple methods including physiochemical, biochemical, immunological, and genetic analyses. Taking account of comprehensive present findings, our isolated materials from the conditioned medium of SHED were determined to EVs, as below: SHED-EVs were shown to be 57–272 nm in diameter with a mean of 86 ± 2.5 nm (means ± SEM), as determined using particle tracking analysis (Fig. 1a). The concentration of SHED-EVs in the CM was 1.6 × 10^9^ ± 4.8 × 10^8^ particles/mL. Moreover, SHED-EVs significantly expressed exosome markers, such as CD9 (83.3 ± 2.5%), CD63 (42.8 ± 3.3%), and CD81 (85.9 ± 6.2%), and reduced expression of the MSC surface marker CD90 (4.1 ± 1.8%), as detected by FCM (Fig. 1b). Western blotting showed an enrichment of CD63 and CD81 in the SHED-EVs; however, the same was not observed for calnexin, which is an endoplasmic reticulum marker, as compared to that SHED (Fig. 1c). We also detected miRNAs and small RNAs within SHED-EVs (the concentrations of miRNAs and small RNAs were 2.8 ± 0.46 ng/μL and 4.6 ± 0.83 ng/μL, respectively; the proportion of miRNA content in the small RNA was 61.1 ± 2.4%; Fig. 1d). The total protein content of SHED-EVs was 828.3 ± 65.8 μg/mL. Next, we depleted the small RNA content, especially miRNAs, of SHED-EVs by treating with RNase (5 U/mL) for 3 h at 37 °C (resulting concentrations of miRNAs and small RNAs were 1.5 ± 0.43 ng/μL and 2.3 ± 0.28 ng/μL, respectively; the proportion of miRNA content in the small RNA was 62.8 ± 3.8%; Fig. 1e). The RNase treatment did not affect the particle size of the SHED-EVs (Fig. 1f). This RNase-treated SHED-EVs expressed the similar phenotypes of membrane surface and total protein content to intact SHED-EVs (data not shown). These findings indicate that SHED-EVs contained enriched RNAs, especially small RNAs, and suggested that the present RNase treatment is useful to assess the efficacy of RNA contents in SHED-EVs.

**Fig. 2:**
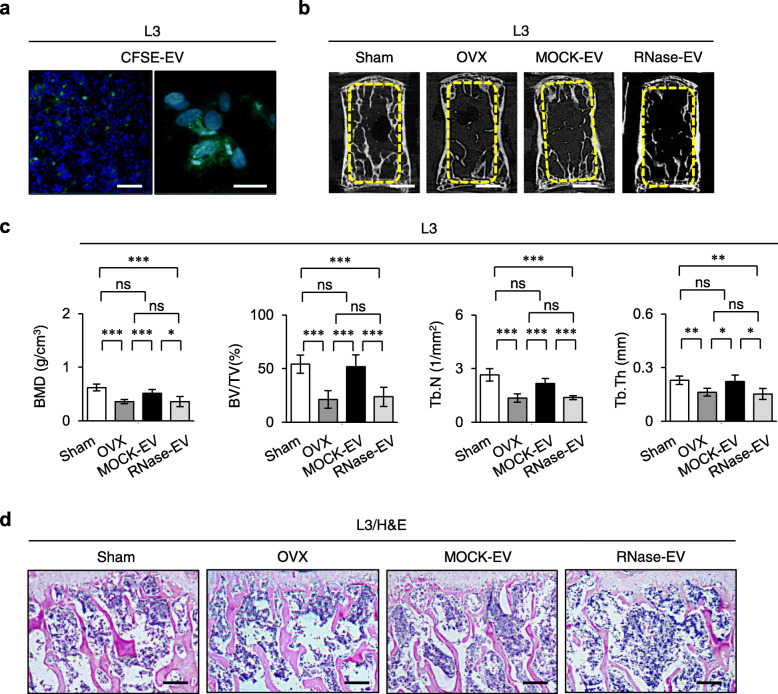
Systemic SHED-EV infusion improved bone loss and enhanced osteoclast activity in recipient ovariectomized (OVX) mice. **a** OVX mice were intravenously administered SHED-EVs (100 μg/mouse) pretreated with carboxyfluorescein diacetate succinimidyl ester (CFSE) 2 days post-surgery. Representative fluorescent micrographs of the bone marrow of OVX mice 3 days after SHED-EV infusion as seen by the cell tracking assay using CFSE-labeled SHED-EVs (CSFE-EVs). Nuclei were stained with 4′,6-diamidino-2-phenylindole (DAPI). Bars = 100 μm. **b**–**d** OVX mice were intravenously administered SHED-EVs (100 μg/mouse) pretreated with or without RNase for 30 min 2 days post-surgery and harvested 4 weeks after infusion. Representative trabecular bone structure of the third lumbar vertebra (L3) by the micro-computed tomography assay (**b**). The graphs show BMD, BV/TV, Tb. N, and Tb. Th of the trabecular bone in L3. *n* = 7 for all groups. Comparisons between two groups were analyzed by an independent two-tailed Student’s *t* test. Multiple group comparisons were performed by a one-way analysis of variance followed by Tukey’s post hoc test. *P < 0.05, **P < 0.01, ***P < 0.005, ns no significance. Graph bars represent mean ± standard deviation (SD) (**c**). Representative trabecular bone structure of L3 by histological assay using hematoxylin and eosin staining (H&E) (**d**). **b**–**d** Sham, sham-operated group; OVX, PBS-infused OVX group; MOCK-EV, MOCK-treated SHED-EV-infused OVX group; RNase-EV, RNase-retreated SHED-EV-infused OVX group. **a**, **b**, **d** Bars = 100 μm (**a**, left), 20 μm (**a**, right), 1 mm (**b**), 200 μm (**d**)

Next, to determine whether EVs released from SHED are an alternative to SHED themselves in postmenopausal osteoporosis therapy, we intravenously infused SHED-EVs (100 μg/mouse) into OVX mice 2 days post-surgery, as referred to the present SHED transplantation into OVX mice. The dose of SHED-EVs was determined based on previous studies [38, 39]. We firstly examined the distribution of SHED-EVs in the bone marrow of OVX mice 3 days after the administration. CFSE-labeled SHED-EVs were up-taken into the recipient bone marrow cells of the recipient bone and bone marrow of OVX mice (Fig. 2a).

After 4 weeks of the systemic SHED-EV administration, the osteoporotic phenotype of OVX mice was observed to be rescued as indicated by the marked increase in BMD, BV/TV, Tb. N, and Tb. Th by micro-CT and histological analyses (Fig. 2b–d). The enhanced in vivo and in vitro osteoclast activity in OVX mice was also rescued 4 weeks after administering SHED-EVs, as indicated by the decrease in the TRAP-positive cells and serum levels of sRANKL and CTX (Additional file 1: Supplementary Figure 7a, 7b) as well as reduced TRAP-positive MNCs and mRNA levels of *Tnfrsf11a*, *Nfatc1*, and *Ctsk* (Additional file 1: Supplementary Figures 8a, 8b). Moreover, to investigate whether RNAs within SHED-EVs play a role in their therapeutic effect, we systemically infused SHED-EVs pretreated with RNase for 3 h at 37 °C into OVX mice and examined the osteoporotic phenotype after 4 weeks. RNase-pretreated SHED-EVs did not contribute to recovering bone reduction and inhibiting osteoclast-mediated bone resorption in OVX mice (Fig. 2b–d, Additional file 1: Supplementary Figures 7, 8). These findings indicate that RNAs within SHED-EVs contribute to the recovery of bone reduction in OVX mice.

### Systemic administration of SHED-EVs rescue impaired BMMSC function via telomerase activity in recipient OVX mice

**Fig. 3:**
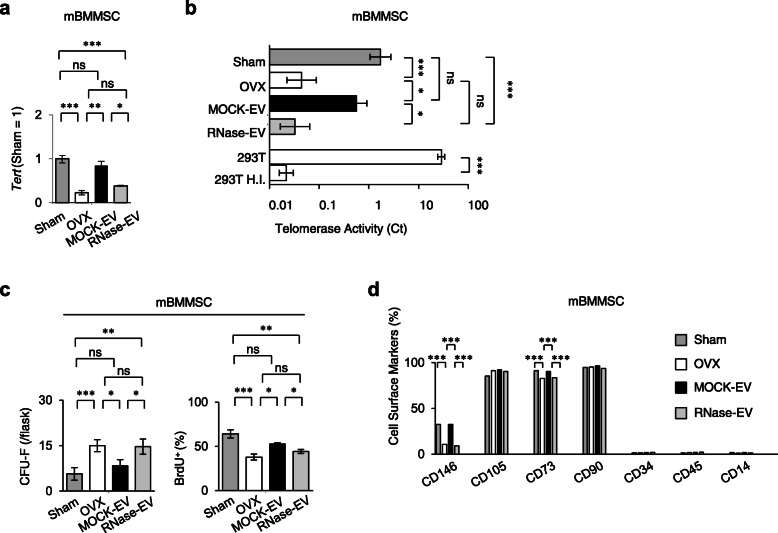
Systemic SHED-EV infusion rescued the properties and functions of mouse bone marrow mesenchymal stem cells (mBMMSCs) in recipient OVX mice. **a** The ratio of the expression of *Tert* in mBMMSCs by reverse transcription-quantitative polymerase chain reaction (RT-qPCR). The data are shown as a ratio to the expression in the mBMMSCs derived from sham-operated mouse (Sham = 1). **b** Telomerase activity in mBMMSCs represented by their Ct values using the RQ-TRAP assay. 293T, HEK 293T cells; 293T H.I., heat-inactivated HEK 293T cells. **c** Number of adherent colonies and proportion of bromodeoxyuridine-positive (BrdU^+^) cells as detected by the CFU-F and BrdU incorporate assays. **d** Proportion of cells positive for each surface marker in mBMMSCs as determined by FCM analysis. **a**–**d** Sham, sham-operated group; OVX, PBS-infused OVX group; MOCK-EV, MOCK-treated SHED-EV-infused OVX group; RNase-EV, RNase-retreated SHED-EV-infused OVX group. *n* = 7 for all groups. Comparisons between two groups were analyzed by an independent two-tailed Student’s *t* test. Multiple group comparisons were performed by a one-way analysis of variance followed by Tukey’s post hoc test.*P < 0.05, **P < 0.01, ***P < 0.005, ns no significance. Graph bars represent mean ± SEM

We next examined the effect of single SHED-EV infusion on impaired BMMSCs in recipient OVX mice and found that recipient BMMSCs recovered *Tert* mRNA levels and telomerase activity, as determined via RT-qPCR and RQ-TRAP, respectively (Fig. 3a, b). However, the administration of RNase-treated SHED-EVs did not rescue the *Tert* mRNA levels and telomerase activity in recipient BMMSCs (Fig. 3a, b).

**Fig. 4:**
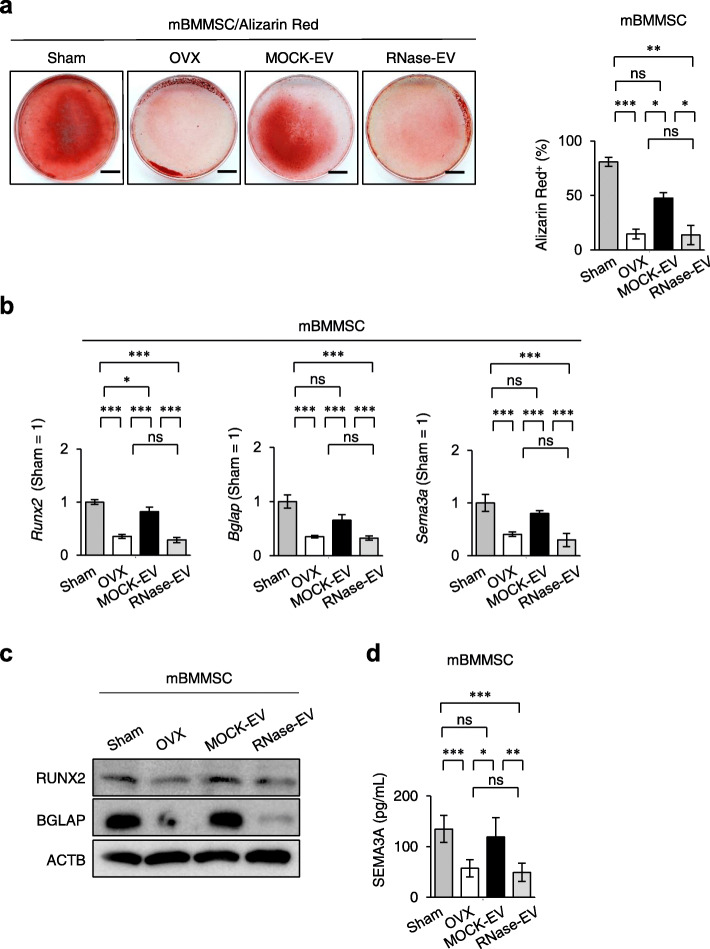
Systemic SHED-EV infusion rescued the functions of mBMMSCs in recipient OVX mice. **a** Representative images of mineralized nodules after Alizarin Red staining 4 weeks after osteogenesis. The Alizarin Red^+^ cells. Bars = 10 mm. The graph shows Alizarin Red-positive (Alizarin Red^+^) areas. **b** The ratio of the expression of osteoblast markers, including *Runt-related transcription factor 2* (*Runx2*) and *bone gamma carboxyglutamic acid protein* (*Bglap*), and osteoprotective marker *semaphorin-3a* (*Sema3a*) by RT-qPCR 1 week after the induction of osteogenesis. The data are shown as a ratio to the expression in the mBMMSCs derived from sham-operated mouse (Sham = 1). **c** The expression of osteoblast markers, including RUNX2 and BGLA by Western blot analysis 1 week after the induction of osteogenesis. ACTB actin, beta. **d** The expression of SEMA3A in the conditioned medium by enzyme-linked sorbent immunoassay (ELISA) 1 week after the induction of osteogenesis. **a**–**d** Sham, sham-operated group; OVX, PBS-infused OVX group; MOCK-EV, MOCK-treated SHED-EV-infused OVX group; RNase-EV, RNase-retreated SHED-EV-infused OVX group. *n* = 7 for all groups. Comparisons between two groups were analyzed by an independent two-tailed Student’s *t* test. Multiple group comparisons were performed by a one-way analysis of variance followed by Tukey’s post hoc test.*P < 0.05, **P < 0.01, ***P < 0.005, ns no significance. Graph bars represent mean ± SEM

Furthermore, the SHED-EV administration improved the impaired stemness, including CFU-F formation, cell proliferation, and expression of CD146 and CD73, of recipient BMMSCs, as detected by the CFU-F, BrdU-labeling, and FCM assays, respectively (Fig. 3c, d). Infusion of SHED-EVs also markedly rescued the impaired function of recipient BMMSCs, as indicated by the increase in mineralized nodule formation and mRNA levels of *Runx2*, *Bglap*, and *Sema3a* by Alizarin Red staining and RT-qPCR under osteogenic conditions (Fig. 4a, b). Western blot analysis and ELISA showed the recovery of protein levels of RUNX2 and BGLAP in recipient BMMSCs (Fig. 4c) and SEMA3A in the conditioned medium of recipient BMMSCs, respectively (Fig. 4d). However, the administration of RNase-treated SHED-EVs did not rescue the stemness and function of recipient BMMSCs (Figs. 3c, d, and 4). These findings indicate that RNAs within SHED-EVs contribute to the recovery of the impaired function of recipient BMMSCs via telomerase activity.

### SHED-EVs ameliorate the properties of hBMMSCs

**Fig. 5:**
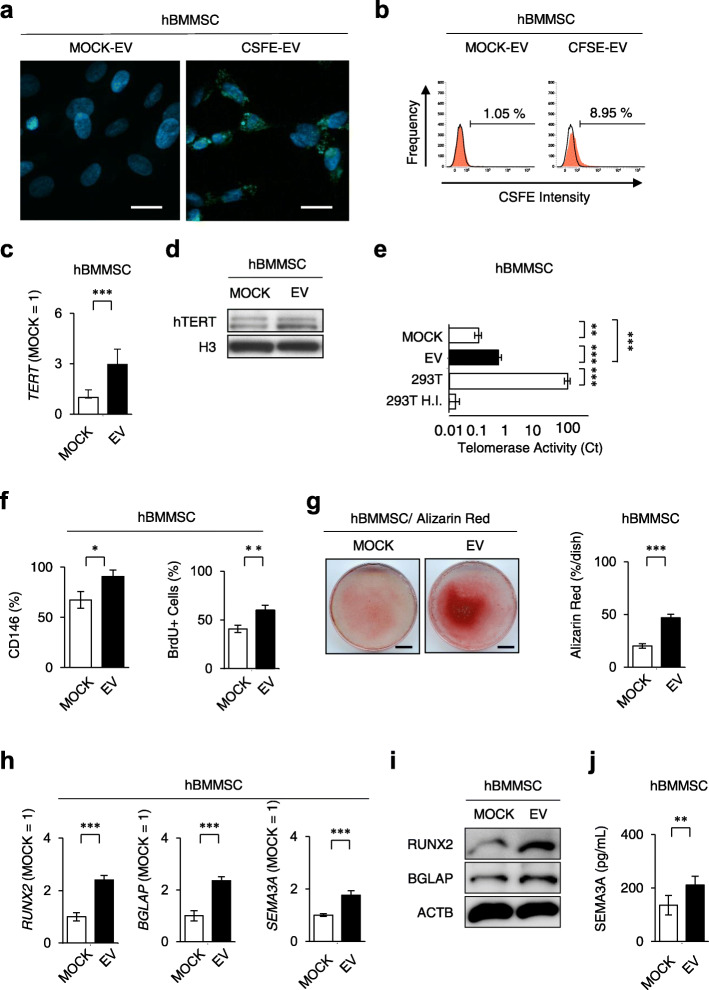
SHED-EVs enhanced Tert-associated telomerase activity and osteogenic functions of human BMMSCs (hBMMSCs). hBMMSCs were incubated with SHED-EVs (EV; 20 μg) or PBS (MOCK) for 3 days. **a**, **b** hBMMSCs were incubated with SHED-EVs loaded with SHED-EVs labeled with CFSE and PBS, CSFE-EVs, and MOCK-EVs, respectively, for 3 days. Representative fluorescent images of hBMMSCs are shown. Nuclei were stained with DAPI (**a**). Representative histograms of hBMMSCs loaded with CFSE-EV and MOCK-EV by FCM analysis. Numbers indicate the means of the positive cells (**b**). **c** Expression of human *TERT* in hBMMSCs by RT-qPCR. **d** Expression of human TERT in hBMMSCs by Western blot analysis. H3 histone 3. **e** Telomerase activity in hBMMSCs as detected by RQ-TRAP. 293T, HEK 293T cells; 293T H.I., heat-inactivated HEK 293T cells. **f** Expression of CD146 and proportion of BrdU^+^ cells in hBMMSCs by FCM and BrdU incorporation analyses. **g** Representative images of mineralized nodules after Alizarin Red staining 4 weeks after osteogenesis. Percentage of the Alizarin Red^+^ area in the dish area. **h** Ratio of the expression of *RUNX2*, *BGLAP*, and *SEMA3A* by RT-qPCR 1 week after the induction of osteogenesis. **i** The expression of osteoblast markers, including RUNX2 and BGLAP by Western blot analysis 1 week after the induction of osteogenesis. ACTB actin, beta. **j** The expression of SEMA3A in the conditioned medium by ELISA 1 week after the induction of osteogenesis. **a**–**j** CFSE-EV, CFSE-pretreated SHED-EV-loaded group; MOCK-EV, PBS-pretreated SHED-EV-loaded group. **a**, **g** Bars = 20 μm (**a**), 10 mm (**g**). **b**, **c**, **e**–**h**, **j***n* = 7 for all groups. Comparisons between two groups were analyzed by an independent two-tailed Student’s *t* test. Multiple group comparisons were performed by a one-way analysis of variance followed by Tukey’s post hoc test.*P < 0.05, **P < 0.01, ***P < 0.005. ns no significance. Graph bars represent mean ± SEM. **c**, **h** The results are shown as a ratio to the expression in the MOCK-EV-treated group (MOCK = 1)

To determine the direct effects of SHED-EVs on BMMSCs in mediating cell-cell communication, we incubated hBMMSCs with or without SHED-EVs. Fluorescence microscopic and FCM assays revealed that SHED-EVs were up-taken into hBMMSCs (Fig. 5a, b). SHED-EV-treated hBMMSCs significantly increased the mRNA and protein levels of TERT by RT-qPCR and Western blot analyses, respectively (Fig. 5c, d), and markedly upregulated telomerase activity by RQ-TRAP (Fig. 5e), when compared to MOCK-treated hBMMSCs. FCM and BrdU assays showed that SHED-EVs upregulated the expression of CD146 and cell proliferation, respectively (Fig. 5f). SHED-EVs also enhanced the in vitro functions of hBMMSCs, as indicated by increased mineralized nodule formation and mRNA levels of *RUNX2*, *BGLAP*, and *SEMA3A* as determined by Alizarin Red staining and RT-qPCR under osteogenic conditions (Fig. 5g, h). Western blot analysis and ELISA showed the enhanced levels of RUNX2 and BGLAP in recipient BMMSCs (Fig. 5i) and the increased level of SEMA3A in the conditioned medium of recipient BMMSCs (Fig. 5j), respectively, under osteogenic conditions.

**Fig. 6:**
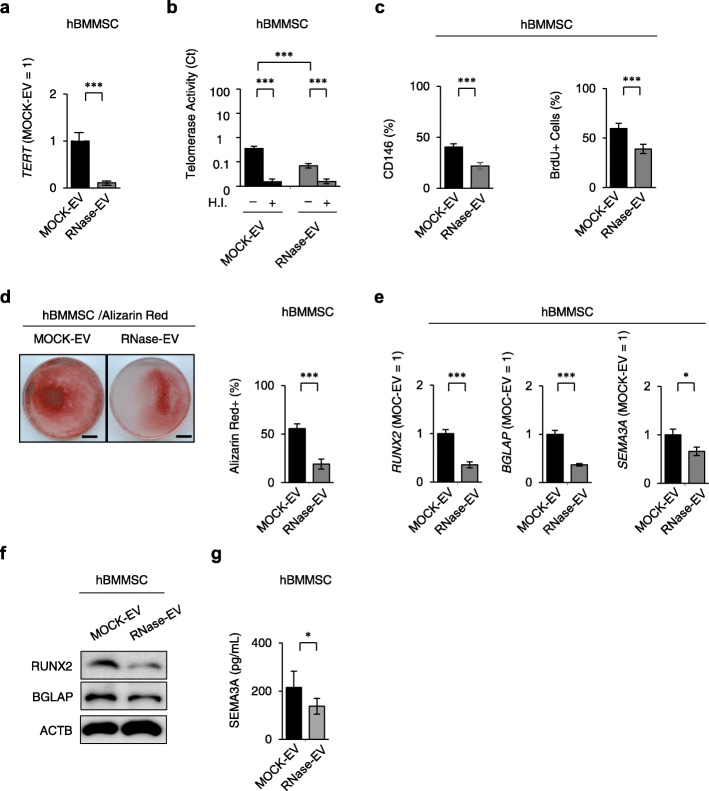
RNAs from hSHED-EVs regulated hSHED-EV-enhanced TRET-associated hBMMSC properties and functions. hBMMSCs were incubated with RNase-pretreated and MOCK-pretreated SHED-EVs (RNase-EV and MOCK-EV, respectively). **a** Expression of *TERT* in hBMMSCs by RT-qPCR. **b** Telomerase activity in hBMMSCs assayed by RQ-TRAP. H.I., heat-inactivated group. **c** Expression of CD146 and percentages of BrdU^+^ cells in hBMMSCs after FCM and BrdU incorporate assays. **d** Representative images of mineralized nodules after Alizarin Red staining 4 weeks after osteogenesis. Bars = 10 mm. Percentage of Alizarin Red^+^ area in dish area. **e** Ratio of the expression of *RUNX2*, *BGLAP*, and *SEMA3A* as detected by RT-qPCR 1 week after the induction of osteogenesis. The results are shown as a ratio to the expression in MOCK-pretreated hSHED-EV-treated group (MOCK-EV = 1, respectively). **f** The expression of osteoblast markers, including RUNX2 and BGLA by Western blot analysis 1 week after the induction of osteogenesis. ACTB actin, beta. **g** The expression of SEMA3A in the conditioned medium by ELISA 1 week after the induction of osteogenesis. **a**–**g** MOCK-EV, MOCK-pretreated hSHED-EV-treated group; RNase-EV, RNase-pretreated hSHED-EV-treated group. *n* = 7 for all groups. Comparisons between two groups were analyzed by an independent two-tailed Student’s *t* test. Multiple group comparisons were performed by a one-way analysis of variance followed by Tukey’s post hoc test.*P < 0.05, ***P < 0.005. ns no significance. Graph bars represent mean ± SEM

Next, to confirm whether RNAs from SHED-EVs regulate hBMMSC properties, we assayed hBMMSCs incubated with RNase-pretreated SHED-EVs. We found that RNase-pretreated SHED-EVs attenuated the SHED-EV-mediated enhancement of *TERT* mRNA levels and telomerase activity (Fig. 6a, b). The RNase pretreatment did not affect the stemness, including CD146 expression and cell proliferation, in hBMMSCs (Fig. 6c). RNase-pretreated SHED-EVs also attenuated SHED-EV-mediated enhancement of function of hBMMSCs under osteogenic condition (Fig. 6d–g). Furthermore, SHED-EV-pretreated hBMMSCs exhibited a significant increase in bone formation when implanted into immunocompromised mice subcutaneously using HA/TCP as a carrier, as indicated by histological analysis (Additional file 1: Supplementary Figure 9a). Immunofluorescent analysis using anti-human BGLAP antibody demonstrated that SHED-EV-pretreatment enhanced bone formation of hBMMSCs (Additional file 1: Supplementary Figure 9b). RNase-pretreated SHED-EVs also attenuated the enhanced de novo bone formation in hBMMSCs (Additional file 1: Supplementary Figures 9a, 9b). These findings indicated that RNAs within SHED-EVs enhanced the function of hBMMSCs via telomerase activity.

Finally, we investigated a mechanism to enhance telomerase activity in BMMSCs by SHED-EV treatment. RT-qPCR showed the expression of MIR346 in SHED-EVs, as well as SHED (Additional file 1: Supplementary Figure 10a). However, RNase pretreatment depleted the expression of *MIR346* in SHED-EVs (Additional file 1: Supplementary Figure 10a). SHED-EV treatment significantly upregulated the expression of MIR346 in hBMMSCs, but RNase pretreatment attenuated the MIR346 expression (Additional file 1: Supplementary Figure 10b).

## Discussion

Postmenopausal osteoporosis, which is the most commonly well-known bone disorder, is caused by marked bone reduction via hyperactivated osteoclasts, leading to a high risk of fragility fractures, but the detailed mechanisms underlying the estrogen-deficient condition are not fully understood [40]. Standard antiresorptive drugs such as bisphosphonates, estrogen-like substrates, and parathyroid hormone effectively prevent bone loss via osteoclasts to decrease the risk of fragility fractures, but they have marked adverse reactions, such as increased risk of cancer, heart disease, and bisphosphonate-related osteonecrosis of the jaw [41, 42], suggesting that novel alternative therapeutic strategy is necessary to treat osteoporosis. Recent studies reveal that systemic infusion of SHED themselves exert therapeutic effects on osteoporosis with multiple mechanisms [3, 12, 13]. Here, we firstly evaluate that systemic administration of SHED-EVs ameliorates the osteoporotic phenotype in OVX mice, especially in the early onset, by rescuing the recipient BMMSC deficiency and inhibiting osteoclast activity. Accumulating studies suggests that MSC-derived EVs might represent specific advantages of no risk of tumor formation and vascular thrombosis, and lower immunogenicity over parent MSCs [43–45]. Therefore, these findings suggest that SHED-EVs have a great advantage in preventing osteoporosis to SHED themselves.

Recent studies show that the major therapeutic benefit of MSC transplantation for osteoporosis in mice employs multiple mechanisms using diverse trophic factors secreted from donor MSCs, but are not imparted by directly replacing of bone-forming cells by engrafted donor MSC-originating matured cells in situ [15, 46]. In SHED-based therapy, cytokine/chemokine secretion and cell-cell interactions of donor cells are proposed to participate in treating osteoporosis [12, 15]. However, it has been unclear whether SHED-EVs participate in SHED-based therapy for osteoporosis. In this study, we firstly demonstrated that the systemic infusion of SHED-EVs improves bone loss in postmenopausal osteoporosis model OVX mice. SHED-EVs also rescued the impaired function of recipient BMMSCs by regulating telomerase activity. Thus, these findings suggest that SHED-EVs may play a crucial role in the biological crosstalk between donor SHED and recipient BMMSCs to achieve therapeutic effects in postmenopausal osteoporosis.

Epigenetic modifications in bone cells affect the development and therapeutic sensitivity of osteoporosis [47]. An MSC transplantation study shows that MSC-derived exosomes transfer FAS to BMMSCs of recipient FAS-mutated MRL/*lpr* mice to modify the DNA methylation status of *Notch*, resulting in the rescue of impaired recipient BMMSC function [15]. In this study, single administration of SHED-EVs continuously recovered the mRNA levels of *Tert* in the recipient BMMSCs in OVX mice, resulting in improved BMMSC function by regulating telomerase activity, but RNase-pretreated SHED-EVs attenuated the effects of SHED-EVs on the recipient BMMSCs in OVX mice. TERT mRNA levels are controlled by epigenetic modifications, including histone acetylation and methylation, in its promoter region under physiological and pathological conditions [48]. Previous studies have shown that estrogen deficiency increases DNA methylation and downregulates telomerase activity in the bone and peripheral blood cells of postmenopausal women and ovariectomized animals [49–51]. Our data suggest that the estrogen-deficient environment in the bone marrow of OVX mice modifies to suppress the expression of TERT mRNA in host BMMSCs via the Tert promoter region. Single administration of SHED-EVs may epigenetically rescue the expression of TERT mRNA in recipient BMMSCs of OVX mice. Additional experiments will be needed to comprehensively examine whether ovariectomy triggers the epigenetic modification of the TERT promoter region and how SHED-EVs rescued the expression of TERT mRNA in recipient BMMSCs of OVX mice.

Multiple miRNAs regulate the function of BMMSCs [52]. For example, microRNA-218-5p targets COL1A1 to promote osteogenic differentiation of OVX-BMMSCs in vitro [53], while microRNA-145 suppresses the osteogenic differentiation of human BMMSCs [54]. In this study, we evaluated that RNase-pretreated SHED-EVs attenuated the SHED-EV-mediated rescue of *Tert*-associated telomerase activity and functions, including cell proliferation, osteogenesis, and SEMA3A secretion, in recipient mBMMSCs of OVX mice and human BMMSCs. Therefore, we speculated that the RNA within SHED-EVs affects TERT-stimulated telomerase activity to increase cell proliferation and osteogenic function of murine and human BMMSCs. To identify the precise miRNA(s) responsible for modifying TERT gene expression in SHED-EVs, we showed that MIR346 expressed in SHED-EVs, as well as the parent SHED. We also demonstrated that SHED-EV treatment enhanced the expression of MIR346 in hBMMSCs. The RNase pretreatment attenuated the expression of MIR346 in SHED-EVs and suppressed SHED-EV-mediated increased expression of MIR346 in hBMMSCs. MIR346 binds to a region in the 3′UTR of TERT mRNA in human cervical cancer cells and astrocytic glioma cells, leading to upregulating TERT expression [55, 56]. TERT is known to accelerate the functions of proliferation and differentiation of BMMSCs [19, 57], while TERT-deficient mice exhibit bone loss via suppressing osteoblasts and accelerating osteoclasts [58]. These findings suggested that SHED-EV-contained MIR346 might participate in rescuing bone reduction in OVX mice via epigenetically regulating TERT mRNA expression in recipient BMMSCs. Since miRNAs are evolutionally conserved [59, 60], murine and human models may share miRNA(s) capable of stimulating *TERT* gene expression in BMMSCs and SHED-EVs. Further study will be necessary to evaluate the epigenetic mechanism to regulate TERT mRNA via SHED-EV-derived MIR346 in recipient BMMSCs.

MSC-secreted exosomes exert therapeutic effects on cell-cell communications in multiple ways [46, 61]. In this study, we identified that SHED-EV-infusion rescued *Sema3a* mRNA levels in BMMSCs of recipient OVX mice. SEMA3A is an osteoprotective factor produced by osteoblasts [62] and inhibits osteoclast differentiation, while promoting osteoblastic bone formation, leading to the recovery of bone volume in OVX mice. Further, overexpression of *Sema3A* mRNA promotes cell proliferation and osteogenic differentiation of BMMSCs [63]. Serum levels of SEMA3A are decreased with age or after menopause in humans [64], suggesting a novel mechanism that osteoblast-mediated communication via SEMA3A plays an important role in rescuing bone reduction by osteoclasts in postmenopausal osteoporotic condition. The present single administration of SHED-EVs shortly after OVX surgery is considered to impact on suppressing osteoclast-mediated bone resorption rather than promoting osteoblast-mediated bone formation. Therefore, our findings propose that SHED-EV-mediated epigenetic modification in recipient BMMSCs of OVX mice continuously employs a preventive approach for rescuing bone reduction in estrogen-deficient condition; RNAs, such as miRNAs, within SHED-EVs epigenetically modified TERT mRNA to rescues telomerase activity, leading to recover the impaired potency of recipient BMMSCs into osteoblasts to secret SEMA3A into the bone microenvironment. This secondary factor then contributes to suppress osteoclast-mediated bone resorption in the estrogen-deficient condition.

A particular cell population, BMMSCs, which are distinguished by cell surface markers including CD105, CD90, and CD73, are considered to specifically contribute to bone regeneration and microenvironment reconstruction [65]. These markers are considered minimum required markers for MSCs, but they are not recognized critical markers for MSCs [30]. CD146-positive MSC population exhibits as crucial self-renewing osteoprogenitor cells to participate in active de novo formation of the bone and bone marrows in vivo to establish hematopoietic stem cells niche [33]. In addition, the reduced CD73 expression contributes to bone loss in osteoporotic OVX mice associated with extracellular adenosine levels [66]. Our study showed that recipient BMMSCs of OVX mice exhibited the reduced function associated with the reduction of CD146 and CD73 expression, but not CD105 and CD90 expression, indicating that osteoporotic condition impaired stemness and function of recipient BMMSCs as reported before [14, 29]. Interestingly, the present SHED-EV administration into OVX mice rescued the CD146 and CD73 expression in recipient MSCs and improved the reduced bone phenotype. These findings suggested that CD146 and CD73 might be responsible markers to assess stem cell property and bone-forming capacity of recipient BMMSCs in a disease condition in the bone marrow such as osteoporosis.

Recent SHED transplant studies have shown that trophic factor secretion and cell-cell interactions are responsible for immune therapy against osteoporosis [12, 15]. Here, we focused on impaired recipient BMMSCs as a therapeutic target and showed that systemic SHED-EV-infusion rescued impaired recipient BMMSCs by regulating TERT-mediated telomerase activity upon transferring RNAs via SHED-EVs. Since SHED-EVs contain a variety of RNAs, such as miRNAs, that are important in systemic cell-cell communication, suggesting that multiple RNAs contribute to the therapeutic potential for osteoporosis in the recipient. However, we cannot exclude the off-target effects exerted on other types of cells in the bone, bone marrow, and other tissues and organs. Hence, additional experiments are required to examine whether SHED-EV infusion induces off-target effects in other cells in OVX mice.

## Conclusions

Taken together, this study demonstrates that systemic SHED-EV infusion achieved therapeutic efficacy in postmenopausal osteoporosis by targeting recipient BMMSCs and epigenetically rescuing telomerase activity by trophic factor(s), specifically miRNA, within the EVs, resulting in bone regeneration. We have revealed the important interaction between telomerase activity and recipient BMMSCs via SHED-released EVs. Our findings expand the current understanding on the mechanism of SHED-based therapy via SHED-secreted EVs and provide new insights into EV-mediated cell-cell communication in SHED-based therapy. Additional investigation is necessary to determine the effector miRNA(s) in SHED-EVs and their epigenetic modification required to rejuvenate impaired recipient BMMSCs by controlling telomerase activity.

## Supplementary information

**Additional file 1 : Supplementary Methods. Supplementary Table 1.** Specific antibodies for flow cytometry and western blotting. **Supplementary Table 2.** TaqMan probes used for the mouse genes. **Supplementary Table 3.** TaqMan probes used for the human genes. **Supplementary Figure 1.** Systemic transplantation of stem cells from human exfoliated deciduous teeth (SHED) rescued the properties and functions of mouse bone marrow mesenchymal stem cells (mBMMSCs) in recipient ovariectomized (OVX) mice. **Supplementary Figure 2.** Systemic transplantation of SHED rescued the telomerase activity and Tert gene expression of mBMMSCs in recipient OVX mice. **Supplementary Figure 3.** Systemic SHED transplantation improved bone loss in OVX mice. **Supplementary Figure 4.** Systemic SHED transplantation reduced the enhanced osteoclast activity in OVX mice. **Supplementary Figure 5.** Systemic SHED transplantation suppressed in vitro osteoclast differentiation of OVX mouse-derived bone marrow cells (BMCs). **Supplementary Figure 6.** Systemic transplanted SHED engrafted in the bone marrow of OVX mice. **Supplementary Figure 7.** Systemic administration of SHED-released extracellular vesicles (SHED-EVs) reduced the enhanced osteoclast activity in OVX mice. **Supplementary Figure 8.** Systemic SHED-EVs administration suppressed in vitro osteoclast differentiation of OVX mouse-derived bone marrow cells (BMCs). **Supplementary Figure 9.** SHED-EVs enhanced in vivo bone formation of human BMMSCs (hBMMSCs). **Supplementary Figure 10.** SHED-EVs express *MIR346* and upregulate the expression of *MIR346* in human bone marrow mesenchymal stem cells (hBMMSCs) after SHED-EV treatment.

## Data Availability

All data generated and analyzed during this study are included in this published article and its supplementary information files.

## Abbreviations

Bglap: Bone gamma carboxyglutamic acid protein
BMD: Bone mineral density
BMMSCs: Bone marrow mesenchymal stem cells
BrdU: Bromodeoxyuridine
BV/TV: Trabecular bone volume versus total volume
CFSE: Carboxyfluorescein diacetate succinimidyl ester
CFU-F: Colony forming units-fibroblast
CM: Conditioned medium
Ctsk: Cathepsin K
CTX: C-terminal telopeptides of type I collagen
ELISA: Enzyme-linked immunosorbent assay
EVs: Extracellular vesicles
FCM: Flow cytometry
HA/TCP: Hydroxyapatite tricalcium phosphate
hBMMSCs: Human bone marrow mesenchymal stem cells
L3: Third lumbar vertebrae
mBMMSCs: Mouse bone marrow mesenchymal stem cells
micro-CT: Micro-computed tomography
miRNAs: MicroRNAs
MNCs: Multinuclear cells
MSCs: Mesenchymal stem cells
Nfatc1: Nuclear factor of activated T cell
OVX-BMMSCs: BMMSCs derived from OVX mice
OVX: Ovariectomy-induced
PBS: Phosphate-buffered saline
Rank: Receptor activator for nuclear factor κB
Rnase: Ribonuclease A
RQ-TRAP: Quantitative telomerase repeated amplification protocol
RT-qPCR: Reverse transcription-quantitative polymerase chain reaction
Runx2: Runt-related transcription factor 2
Sema3a: Semaphorin-3a
SHED: Stem cells from human exfoliated deciduous teeth
SLE: Systemic lupus erythematosus
SHED-BMMSCs: SHED-transplanted mice-derived bone marrow mesenchymal stem cells
SHED-EVs: SHED-releasing extracellular vesicles
sRANKL: Soluble receptor activator for nuclear factor κB ligand
Tb.N: Trabecular numbers
Tb.Th: Trabecular thickness
TERT: Telomerase reverse transcriptase
TRAP: Tartrate-resistant acid phosphatase

## References

[1] Miura M, Gronthos S, Zhao M, Lu B, Fisher LW, Robey PG (2003). SHED: stem cells from human exfoliated deciduous teeth. Proc Natl Acad Sci U S A.

[2] Fujiyoshi J, Yamaza H, Sonoda S, Yuniartha R, Ihara K, Nonaka K (2019). Therapeutic potential of hepatocyte-like-cells converted from stem cells from human exfoliated deciduous teeth in fulminant Wilson’s disease. Sci Rep.

[3] Yamaza T, Kentaro A, Chen C, Liu Y, Shi Y, Gronthos S (2010). Immunomodulatory properties of stem cells from human exfoliated deciduous teeth. Stem Cell Res Ther.

[4] Shen WC, Lai YC, Li LH, Liao K, Lai HC, Kao SY (2019). Methylation and PTEN activation in dental pulp mesenchymal stem cells promotes osteogenesis and reduces oncogenesis. Nat Commun.

[5] Xuan K, Li B, Guo H, Sun W, Kou X, He X (2018). Deciduous autologous tooth stem cells regenerate dental pulp after implantation into injured teeth. Sci Transl Med.

[6] Yamaza T, Alatas FS, Yuniartha R, Yamaza H, Fujiyoshi JK, Yanagi Y (2015). In vivo hepatogenic capacity and therapeutic potential of stem cells from human exfoliated deciduous teeth in liver fibrosis in mice. Stem Cell Res Ther.

[7] Sakai K, Yamamoto A, Matsubara K, Nakamura S, Naruse M, Yamagata M (2012). Human dental pulp-derived stem cells promote locomotor recovery after complete transection of the rat spinal cord by multiple neuro-regenerative mechanisms. J Clin Invest.

[8] Taguchi T, Yanagi Y, Yoshimaru K, Zhang XY, Matsuura T, Nakayama K (2019). Regenerative medicine using stem cells from human exfoliated deciduous teeth (SHED): a promising new treatment in pediatric surgery. Surg Today.

[9] Matsubara K, Matsushita Y, Sakai K, Kano F, Kondo M, Noda M (2015). Secreted ectodomain of sialic acid-binding Ig-like lectin-9 and monocyte chemoattractant protein-1 promote recovery after rat spinal cord injury by altering macrophage polarity. J Neurosci.

[10] Hirata M, Ishigami M, Matsushita Y, Ito T, Hattori H, Hibi H (2016). Multifaceted therapeutic benefits of factors derived from dental pulp stem cells for mouse liver fibrosis. Stem Cells Transl Med.

[11] Ishikawa J, Takahashi N, Matsumoto T, Yoshioka Y, Yamamoto N, Nishikawa M (2016). Factors secreted from dental pulp stem cells show multifaceted benefits for treating experimental rheumatoid arthritis. Bone..

[12] Ma L, Aijima R, Hoshino Y, Yamaza H, Tomoda E, Tanaka Y (2015). Transplantation of mesenchymal stem cells ameliorates secondary osteoporosis through interleukin-17-impaired functions of recipient bone marrow mesenchymal stem cells in MRL/lpr mice. Stem Cell Res Ther.

[13] Liu Y, Wang L, Liu S, Liu D, Chen C, Xu X (2014). Transplantation of SHED prevents bone loss in the early phase of ovariectomy-induced osteoporosis. J Dent Res.

[14] Sun L, Akiyama K, Zhang H, Yamaza T, Hou Y, Zhao S (2009). Mesenchymal stem cell transplantation reverses multiorgan dysfunction in systemic lupus erythematosus mice and humans. Stem Cells.

[15] Liu S, Liu D, Chen C, Hamamura K, Moshaverinia A, Yang R (2015). MSC transplantation improves osteopenia via epigenetic regulation of notch signaling in lupus. Cell Metab.

[16] Blackburn EH (2005). Telomeres and telomerase: their mechanisms of action and the effects of altering their functions. FEBS Lett.

[17] Poole JC, Andrews LG, TO T (2001). Activity, function, and gene regulation of the catalytic subunit of telomerase (hTERT). Gene..

[18] Flores I, Benetti R, Blasco MA (2006). Telomerase regulation and stem cell behaviour. Curr Opin Cell Biol.

[19] Shi S, Gronthos S, Chen S, Reddi A, Counter CM, Robey PG (2002). Bone formation by human postnatal bone marrow stromal stem cells is enhanced by telomerase expression. Nat Biotechnol.

[20] Simonsen JL, Rosada C, Serakinci N, Justesen J, Stenderup K, Rattan SIS (2002). Telomerase expression extends the proliferative life-span and maintains the osteogenic potential of human bone marrow stromal cells. Nat Biotechnol.

[21] Liu Y, Wang L, Kikuiri T, Akiyama K, Chen C, Xu X (2011). Mesenchymal stem cell-based tissue regeneration is governed by recipient T lymphocytes via IFN-γ and TNF-α. Nat Med.

[22] Chen C, Akiyama K, Yamaza T, You YO, Xu X, Li B (2014). Telomerase governs immunomodulatory properties of mesenchymal stem cells by regulating FAS ligand expression. EMBO Mol Med.

[23] Bruno S, Kholia S, Deregibus MC, Camussi G (2019). The role of extracellular vesicles as paracrine effectors in stem cell-based therapies. Adv Exp Med Biol.

[24] Phinney DG, Di Giuseppe M, Njah J, Sala E, Shiva S, St Croix CM (2015). Mesenchymal stem cells use extracellular vesicles to outsource mitophagy and shuttle microRNAs. Nat Commun.

[25] Jarmalavičiute A, Tunaitis V, Pivoraite U, Venalis A, Pivoriunas A (2015). Exosomes from dental pulp stem cells rescue human dopaminergic neurons from 6-hydroxy-dopamine-induced apoptosis. Cytotherapy..

[26] Pivoraitė U, Jarmalavičiūtė A, Tunaitis V, Ramanauskaitė G, Vaitkuvienė A, Kašėta V (2015). Exosomes from human dental pulp stem cells suppress carrageenan-induced acute inflammation in mice. Inflammation..

[27] Li Y, Yang YY, Ren JL, Xu F, Chen FM, Li A (2017). Exosomes secreted by stem cells from human exfoliated deciduous teeth contribute to functional recovery after traumatic brain injury by shifting microglia M1/M2 polarization in rats. Stem Cell Res Ther.

[28] Narbute K, Piļipenko V, Pupure J, Dzirkale Z, Jonavičė U, Tunaitis V (2019). Intranasal administration of extracellular vesicles derived from human teeth stem cells improves motor symptoms and normalizes tyrosine hydroxylase expression in the substantia nigra and striatum of the 6-hydroxydopamine-treated rats. Stem Cells Transl Med.

[29] Yamaza T, Miura Y, Bi Y, Liu Y, Akiyama K, Sonoyama W (2008). Pharmacologic stem cell based intervention as a new approach to osteoporosis treatment in rodents. PLoS One.

[30] Dominici M, Le Blanc K, Mueller I, Slaper-Cortenbach I, Marini FCF, Krause DS (2006). Minimal criteria for defining multipotent mesenchymal stromal cells. The International Society for Cellular Therapy position statement. Cytotherapy..

[31] Ward WE, Kim S, Chan D, Fonseca D (2005). Serum equol, bone mineral density and biomechanical bone strength differ among four mouse strains. J Nutr Biochem.

[32] Zhou H, Iida-Klein A, Lu SS, Ducayen-Knowles M, Levine LR, Dempster DW (2003). Anabolic action of parathyroid hormone on cortical and cancellous bone differs between axial and appendicular skeletal sites in mice. Bone..

[33] Sacchetti B, Funari A, Michienzi S, Di Cesare S, Piersanti S, Saggio I (2007). Self-renewing osteoprogenitors in bone marrow sinusoids can organize a hematopoietic microenvironment. Cell..

[34] Black DM, Rosen CJ (2016). Clinical practice. Postmenopausal Osteoporosis N Engl J Med.

[35] Huang YC, Parolini O, Deng L (2013). The potential role of microvesicles in mesenchymal stem cell-based therapy. Stem Cells Dev.

[36] Katsuda T, Kosaka N, Takeshita F, Ochiya T (2013). The therapeutic potential of mesenchymal stem cell-derived extracellular vesicles. Proteomics..

[37] Tavallaie R, McCarroll J, Le Grand M, Ariotti N, Schuhmann W, Bakker E (2018). Nucleic acid hybridization on an electrically reconfigurable network of gold-coated magnetic nanoparticles enables microRNA detection in blood. Nat Nanotechnol.

[38] Liu S, Liu D, Chen C, Hamamura K, Moshaverinia A, Yang R (2015). MSC transplantation improves osteopenia via epigenetic regulation of notch signaling in lupus. Cell Metab.

[39] Willis GR, Kourembanas S, Mitsialis SA (2017). Toward exosome-based therapeutics: isolation, heterogeneity, and fit-for-purpose potency. Front Cardiovasc Med.

[40] Eastell R, O’Neill TW, Hofbauer LC, Langdahl B, Reid IR, Gold DT (2016). Postmenopausal osteoporosis. Nat Rev Dis Prim.

[41] Delmas PD (2002). Treatment of postmenopausal osteoporosis. Lancet..

[42] Rachner TD, Khosla S, Hofbauer LC (2011). Osteoporosis: now and the future. Lancet..

[43] de Jong OG, van Balkom BWM, Schiffelers RM, Bouten CVC, Verhaar MC (2014). Extracellular vesicles: potential roles in regenerative medicine. Front Immunol.

[44] Qin Y, Sun R, Wu C, Wang L, Zhang C (2016). Exosome: a novel approach to stimulate bone regeneration through regulation of osteogenesis and angiogenesis. Int J Mol Sci.

[45] Abbaszadeh H, Ghorbani F, Derakhshani M, Movassaghpour A, Yousefi M (2020). Human umbilical cord mesenchymal stem cell-derived extracellular vesicles: a novel therapeutic paradigm. J Cell Physiol.

[46] Otsuru S, Desbourdes L, Guess AJ, Hofmann TJ, Relation T, Kaito T (2018). Extracellular vesicles released from mesenchymal stromal cells stimulate bone growth in osteogenesis imperfecta. Cytotherapy..

[47] van Meurs JB, Boer CG, Lopez-Delgado L, Riancho JA (2019). Role of epigenomics in bone and cartilage disease. J Bone Miner Res.

[48] Hanahan D, Weinberg RA (2011). Hallmarks of cancer: the next generation. Cell..

[49] Cen J, Zhang H, Liu Y, Deng M, Tang S, Liu W (2015). Anti-aging effect of estrogen on telomerase activity in ovariectomised rats - animal model for menopause. Gynecol Endocrinol.

[50] Reppe S, Lien TG, Hsu YH, Gautvik VT, Olstad OK, Yu R (2017). Distinct DNA methylation profiles in bone and blood of osteoporotic and healthy postmenopausal women. Epigenetics..

[51] Cheishvili D, Parashar S, Mahmood N, Arakelian A, Kremer R, Goltzman D (2018). Identification of an epigenetic signature of osteoporosis in blood DNA of postmenopausal women. J Bone Miner Res.

[52] Wang J, Liu S, Li J, Zhao S, Yi Z (2019). Roles for miRNAs in osteogenic differentiation of bone marrow mesenchymal stem cells. Stem Cell Res Ther.

[53] Kou J, Zheng X, Guo J, Liu Y, Liu X (2019). MicroRNA-218-5p relieves postmenopausal osteoporosis through promoting the osteoblast differentiation of bone marrow mesenchymal stem cells. J Cell Biochem.

[54] Jin Y, Hong F, Bao Q, Xu Q, Duan R, Zhu Z, et al. MicroRNA-145 suppresses osteogenic differentiation of human jaw bone marrow mesenchymal stem cells partially via targeting semaphorin 3A. Connect Tissue Res. 2019. 10.1080/03008207.2019.1643334. 10.1080/03008207.2019.1643334 31305177

[55] Song G, Wang R, Guo J, Liu X, Wang F, Qi Y (2015). MiR-346 and miR-138 competitively regulate hTERT in GRSF1- and AGO2-dependent manners, respectively. Sci Rep.

[56] Wolter M, Werner T, Malzkorn B, Reifenberger G (2016). Role of microRNAs located on chromosome arm 10q in malignant gliomas. Brain Pathol.

[57] Shi S, Gronthos S (2003). Perivascular niche of postnatal mesenchymal stem cells in human bone marrow and dental pulp. J Bone Miner Res.

[58] Saeed H, Abdallah BM, Ditzel N, Catala-Lehnen P, Qiu W, Amling M (2011). Telomerase-deficient mice exhibit bone loss owing to defects in osteoblasts and increased osteoclastogenesis by inflammatory microenvironment. J Bone Miner Res.

[59] Lee CT, Risom T, Strauss WM (2007). Evolutionary conservation of microRNA regulatory circuits: an examination of microRNA gene complexity and conserved microRNA-target interactions through metazoan phylogeny. DNA Cell Biol.

[60] Berezikov E (2011). Evolution of microRNA diversity and regulation in animals. Nat Rev Genet.

[61] Bernardo ME, Fibbe WE (2013). Mesenchymal stromal cells: sensors and switchers of inflammation. Cell Stem Cell.

[62] Hayashi M, Nakashima T, Taniguchi M, Kodama T, Kumanogoh A, Takayanagi H (2012). Osteoprotection by semaphorin 3A. Nature..

[63] Liu L, Wang J, Song X, Zhu Q, Shen S, Zhang W (2018). Semaphorin 3A promotes osteogenic differentiation in human alveolar bone marrow mesenchymal stem cells. Exp Ther Med.

[64] Hayashi M, Nakashima T, Yoshimura N, Okamoto K, Tanaka S, Takayanagi H (2019). Autoregulation of osteocyte Sema3A orchestrates estrogen action and counteracts bone aging. Cell Metab.

[65] Kfoury Y, Scadden DT (2015). Mesenchymal cell contributions to the stem cell niche. Cell Stem Cell.

[66] Shih YRV, Liu M, Kwon SK, Iida M, Gong Y, Sangaj N (2019). Dysregulation of ectonucleotidase-mediated extracellular adenosine during postmenopausal bone loss. Sci Adv.

