# Supplementary material for: Extracellular vesicles from deciduous pulp stem cells recover bone loss by regulating telomerase activity in an osteoporosis mouse model
Source: Stem Cell Res Ther. 2020 Jul 17;11:296. doi: 10.1186/s13287-020-01818-0 (PMC7367365; doi:10.1186/s13287-020-01818-0)
Supplement: Supplementary file 1 — Additional file 1 : Supplementary Methods. Supplementary Table 1. Specific antibodies for flow cytometry and western blotting. Supplementary Table 2. TaqMan probes used for the mouse genes. Supplementary Table 3. TaqMan probes used for the human genes. Supplementary Figure 1. Systemic transplantation of stem cells from human exfoliated deciduous teeth (SHED) rescued the properties and functions of mouse bone marrow mesenchymal stem cells (mBMMSCs) in recipient ovariectomized (OVX) mice. Supplementary Figure 2. Systemic transplantation of SHED rescued the telomerase activity and Tert gene expression of mBMMSCs in recipient OVX mice. Supplementary Figure 3. Systemic SHED transplantation improved bone loss in OVX mice. Supplementary Figure 4. Systemic SHED transplantation reduced the enhanced osteoclast activity in OVX mice. Supplementary Figure 5. Systemic SHED transplantation suppressed in vitro osteoclast differentiation of OVX mouse-derived bone marrow cells (BMCs). Supplementary Figure 6. Systemic transplanted SHED engrafted in the bone marrow of OVX mice. Supplementary Figure 7. Systemic administration of SHED-released extracellular vesicles (SHED-EVs) reduced the enhanced osteoclast activity in OVX mice. Supplementary Figure 8. Systemic SHED-EVs administration suppressed in vitro osteoclast differentiation of OVX mouse-derived bone marrow cells (BMCs). Supplementary Figure 9. SHED-EVs enhanced in vivo bone formation of human BMMSCs (hBMMSCs). Supplementary Figure 10. SHED-EVs express MIR346 and upregulate the expression of MIR346 in human bone marrow mesenchymal stem cells (hBMMSCs) after SHED-EV treatment. [file 13287_2020_1818_MOESM1_ESM.docx]

**Supplementary Information**

**Extracellular vesicles from deciduous pulp stem cells recover bone loss by regulating telomerase activity in an osteoporosis mouse model**

Soichiro Sonoda, Sara Murata, Kento Nishida, Hiroki Kato, Norihisa Uehara, Yukari N. Kyumoto, Haruyoshi Yamaza, Ichiro Takahashi, Toshio Kukita, Takayoshi Yamaza

**Supplementary Methods**

**Isolation and culture of stem cells from human exfoliated deciduous teeth (SHED) and human bone marrow mesenchymal stem cells (hBMMSCs)**

SHED were separately isolated from three different healthy pediatric donors according to the previous reports previously [1–4]. Briefly, remnant dental pulp tissues from human deciduous teeth were digested with 0.3% collagenase type I (Worthington Biochemicals, Lakewood, NJ, USA) and 0.4% dispase II (Sanko Junyaku, Tokyo, Japan) for 60 min at 37 °C and passed through a 70-µm cell strainer. Human bone marrow (AllCells, Alameda, CA) was used to separate the leukocyte fraction using Ficoll-Paque PREMIUM (GE Healthcare, Chicago, IL) according to the manufacturer’s protocol and passed through a 70-µm cell strainer. Single suspensions comprising all nuclear cells (ANCs) from dental pulp tissues and bone marrow were seeded in T-75 culture flasks, which were washed with Ca^2+^ and Mg^2+^-free phosphate buffered saline (PBS, pH 7.4) and non-adherent cells were removed after 24 h incubation. Cells attached were maintained at 37 °C with 5% CO_2_ for 14 days in a growth medium consisting of 15% fetal bovine serum (FBS; Equitech-Bio, Kerrville, TX), 100 µM L-ascorbic acid 2-phosphate (Wako Pure Chemicals, Osaka, Japan), 2 mM L-glutamine (Nacalai Tesque, Kyoto, Japan), and premixed antibiotics containing 100 U/ml penicillin and 100 µg/mL streptomycin (Nacalai Tesque) in Minimum Essential Medium Eagle alpha Modification (αMEM; Thermo Fisher Scientific, Waltham, MA, USA). The present culture method with ascorbic acid was performed according to the previous studies [3–7]. Attached colonies were formed 14 days after seeding and visualized by microscopy. The attached colony-forming cells were passaged five times at 4000 cells/cm^2^. Passage 5 (P5) SHED and hBMMSCs were confirmed by criteria used for mesenchymal stem cells (MSCs) [8], including plastic adherence, surface antigen expression, and multipotency of osteogenic, chondrogenic, and adipogenic differentiation (**data not shown**), according to previous studies [3,4,7], and were used for further experiments.

To collect conditioned medium (CM) from SHED cultures, SHED were cultured in 15% exosome-depleted FBS (Equitech-Bio), 100 µM L-ascorbic acid 2-phosphate (Wako Pure Chemicals), 2 mM L-glutamine (Nacalai Tesque), and premixed antibiotics containing 100 U/mL penicillin and 100 µg/ml streptomycin (Nacalai Tesque) in αMEM (Thermo Fisher Scientific) until 70% confluency was achieved. To deplete FBS-derived EVs, FBS (Equitech-Bio) were centrifuged at 4 °C for 16 h at 10,000 ×*g* using a himmac CP80a ultracentrifuge (Hitachi, Tokyo, Japan) equipped with a P40ST swing rotor (Hitachi) in 13PA tubes (Hitachi).

**Systemic infusion of SHED into mice with postnatal osteoporosis**

Ovariectomized female C57BL/6J mice (10 weeks old; OVX mice) were intravenously administered SHED (0.1 × 10^6^/100 μL of phosphate-buffered saline (PBS)/10 g body weight) two days post-surgery and sacrificed four weeks post-surgery. Age-matched sham-operated C57BL/6J and OVX mice infused with PBS (100 μL/10g body weight) served as experimental controls.

***In vivo tracing assays***

Carboxyfluorescein diacetate succinimidyl ester (CFSE; Thermo Fisher Scientific) or PBS was used for labeling according to the kit instructions. CSFE-SHED (0.1 × 10^6^/100 μL of PBS/10 g body weight) were intravenously infused into OVX mice (10 weeks old) 2 days post-surgery. After 7 days of infusion, prepared frozen sections were mounted using Vectashield mounting medium containing 4′,6-diamidino-2-phenylindole Vector Laboratories, Burlingame, CA, USA).

**Isolation and culture of mouse BMMSCs (mBMMSCs)**

Briefly, mouse bone marrow‐derived ANCs were obtained from the femurs and tibia of mice and seeded at 15 × 10^6^ cells into 100‐mm culture dishes. Non-adherent cells were removed after 48 h. Attached cells were maintained at 37 °C in 5% CO_2_ for 16 days in a mouse growth medium until colonies were formed. The mouse growth medium consisted of 20% FBS (Equitech‐Bio), 2 mM l‐glutamine (Nacalai Tesque), 55 μM 2‐mercaptoethanol (Thermo Fisher Scientific), and premixed antibiotics containing 100 U/mL penicillin and 100 µg/ml streptomycin (Nacalai Tesque) in αMEM (Thermo Fisher Scientific). Attached colony-forming cells were passaged once and P1 mBMMSCs were confirmed as MSCs (**data not shown**) according to a previous study [9] and were used for further experiments.

**Flow cytometric analysis**

Cultured cells (0.1 × 10^6^/100 µL) were resuspended in ice-cold Hanks’s balanced salt solution (Nacalai Tesque) containing 2% heat-inactivated FBS (Equitech-Bio) and incubated with R-phycoerythrin (R-PE)-conjugated primary antibodies (1 µg per antigen) at 4 °C for 45 min. As a control, isotype-matched antibodies conjugated with R-PE were used in place of the corresponding antigen-specific antibody. The stained cells were washed with Hanks’s balanced salt solution containing 2% FBS (Equitech-Bio) and assayed with the FACSVerse flow cytometer (BD Bioscience, Franklin Lake, NJ). The number (percentage) of positive cells was determined using the FACSuite software (BD Bioscience) by comparing with the corresponding control cells stained with the corresponding isotype-matched antibody with a false-positive rate of less than 1% [7]. **Table S1** lists the specific antibodies used in the flow cytometric analysis.

**Western blotting**

Cells were lysed in M‐PER mammalian protein extraction reagent (Thermo Scientific, Waltham, MA, USA) with proteinase inhibitor cocktail (Nacalai Tesque) and phosphatase inhibitor PhoSTOP (Roche); proteins were quantified using the protein concentration assay (Bio‐Rad Laboratories, Hercules, CA, USA). Twenty microgram of total proteins (0.5 µg/20 µL/well) were separated by TGX FastCast acrylamide gels (Bio-Rad Laboratories) and transferred on polyvinylidene fluoride membranes (Bio-Rad Laboratories) using the Trans-Blot Turbo transfer system (Bio-Rad Laboratories). The membranes were blocked with 5% non‐fat dry milk and 0.1% Tween‐20 in Tris-buffered saline (150 mM NaCl and 20 mM Tris–HCl, pH 7.2) for 1 h. The membranes were then incubated with primary antibodies overnight followed by incubation with HRP‐conjugated secondary antibody (1:1,000; Santa Cruz Biotechnology) for 1 h. Immunoreactive proteins were detected using the SuperSignal West Pico Chemiluminescent Substrate (Thermo Fisher Scientific) with the Image Quant LAS 4010 imager (GE Healthcare Life Science, Pittsburgh, PA). For the internal control assay, each membrane was treated with WB Stripping Solution Strong (Nacalai Tesque). The membranes were reprobed with anti-β-actin antibody (Merck, Darmstadt, Germany) followed by incubation with HRP-conjugated secondary antibody (1:1,000; Santa Cruz Biotechnology). **Table S1** lists the specific antibodies used in the flow cytometric analysis.

**Enzyme-linked immunosorbent assay (ELISA)**

Total protein concentration of samples was quantified using the protein concentration assay (Bio‐Rad Laboratories). ELISA were performed using commercially available kits. The ELISA kits used in this study were the RatLaps ELISA (Nordic Bioscience Diagnostics A/S, Herlev, Denmark),Mouse TRANCE/RANKL/TNFSF11 Quantikine ELISA (R&D Systems, Minneapolis, MN), Mouse Semaphorin-3A, SEMA3A ELISA (MyBioSourse, San Diego, CA), Human Semaphorin-3A, SEMA3A ELISA (MyBioSourse) Kits for mouse C-terminal telopeptides of type I collagen (CTX), mouse soluble receptor activator for nuclear factor κB ligand (sRANKL), mouse Semaphorin-3A (SEMA3A), and human SEMA3A, respectively. Finally, the results were measured with the Multiskan FC microplate reader (Thermo Fisher Scientific).

**Reverse transcription and quantitative polymerase chain reaction (RT-qPCR)**

Cells were treated with TRIzol (Thermo Fisher Scientific) and digested with DNase I (Promega, Fichburg, WI) to extract total RNA, which was then purified using the RNeasy Mini Kit (Qiagen, Venlo, Netherland). The gels were stained with ethidium bromide and imaged on a Gel Doc EZ System (Bio-Rad Laboratories). cDNAs was prepared by reverse transcription using the Revertra Ace qPCR kit (TOYOBO, Osaka, Japan) according to the manufacturer instructions. Real-time RT-PCR was performed using the EagleTaq Universal Master Mix (Roche, Basel, Switzerland) with target-specific TaqMan probes (Thermo Fisher Scientific) and the Light Cycler 96 system (Roche). Human 18S ribosomal RNA was used for normalization. **Table S2 and S3** lists the target-specific TaqMan probes used in the qPCR.

**Colony-forming unit-fibroblastic (CFU-F) assays**

All nuclear cells (ANCs; 1 × 10^6^) from mouse bone marrow were seeded into 100-mm culture dishes. After 16 days, the cultures were treated with 2% paraformaldehyde and 1% toluidine blue in PBS. Clusters with more than 50 cells were counted as colonies under a microscope.

**Cell proliferation**

BMMSCs (10 × 10^3^/well) were seeded on 2-well chamber slides (Nunc, Rochester, NY, USA) and incubated with bromodeoxyuridine (BrdU; 1:100; Merck) for 20 h. Cells were stained with a 5-Bromo-2′-deoxy-uridine Labeling and Detection kit I (Merck) according to the manufacturer instructions. BrdU-positive cells were calculated from ten randomly selected images per subject.

***In vivo* osteoclast activity analysis**

Tissue samples were fixed with 4% paraformaldehyde in PBS and decalcified with 10% EDTA followed by preparing paraffin sections. Paraffin sections of bone samples were treated with tartrate-resistant acid phosphatase (TRAP). The sections were pretreated with a mixture of 50% ethanol and 50% acetone for 10 min. Two TRAP staining solutions were freshly mixed: 9.6 mg of naphthol AS-BI phosphate (Merck) in 0.6 mL of N, N-dimethylformamide (Merck), 84 mg of fast red-violet LB diazonium salt (Merck), 58.2 mg of tartaric acid (Merck), and 240 μL of 10% MgCl_2_ in 60 mL of 0.2 M sodium acetate buffer (pH 5.0). The sections were incubated for 10 min at 37 °C under a shield and lightly counterstained with 0.5% toluidine blue. Seven representative images were randomly selected to measure the number of TRAP-positive cells per total bone area in the bone metaphysis using the Image J software (National Institutes of Health, Bethesda, MA). Serum CTX and sRANKL were measured by ELISA.

***In vitro* osteoclast activity analysis**

Mouse bone marrow cells (BMCs; 1 × 10^6^/well) were co-cultured with mouse newborn calvarial osteoblastic cells (1 × 10^5^/well) for 7 days with FBS (Equitech-Bio, Kerrville, TX), 10 nM vitamin D_3_ (Wako Pure Chemical, Osaka, Japan), 1 nM prostaglandin E_2_ (Wako Pure Chemical), and premixed antibiotics containing 100 U/mL penicillin and 100 µg/ml streptomycin (Nacalai Tesque) in αMEM (Thermo Fisher Scientific).The number of TRAP-positive multinucleated cells (> 3 nuclei) were counted using an Acid Phosphatase, Leukocyte (TRAP) Kit (Merck). Osteoclast markers, including receptor activator of nuclear factor kappa B, nuclear factor of activated T-cell (Nfatc1), and cathepsin K were analyzed by RT-qPCR.

***In vivo* osteogenesis**

hBMMSCs (4.0 × 10^6^) were mixed with hydroxyapatite tricalcium phosphate (HA/TCP) particles (40 mg, Zimmer, Warsaw, IN) and subcutaneously implanted under the dorsal skin of immunodeficient Balb/c *nu/nu* mice (female, 10 weeks old). Tissue samples were fixed with 4% paraformaldehyde in PBS and decalcified with 10% EDTA followed by preparing paraffin sections. Paraffin sections were treated with hematoxylin and eosin. Seven representative images were randomly selected to measure the area of newly-formed bone per total bone area using the Image J software (National Institutes of Health). For immunofluorescent microscopy, paraffin sections were incubated with anti-human bone gamma carboxyglutamic acid protein antibody (1:100; MyBioSource, San Diego, CA), followed by incubation with Texas Red-conjugated secondary antibody (1:200; Santa Cruz Biotechnology).

**Supplementary References**

1. Ma L, Aijima R, Hoshino Y, Yamaza H, Tomoda E, Tanaka Y, et al. Transplantation of mesenchymal stem cells ameliorates secondary osteoporosis through interleukin-17-impaired functions of recipient bone marrow mesenchymal stem cells in MRL/lpr mice. Stem Cell Res Ther. 2015;6:104.

2. Sonoda S, Yamaza H, Ma L, Tanaka Y, Tomoda E, Aijima R, et al. Interferon-gamma improves impaired dentinogenic and immunosuppressive functions of irreversible pulpitis-derived human dental pulp stem cells. Sci Rep. 2016;6:19286.

3. Fujiyoshi J, Yamaza H, Sonoda S, Yuniartha R, Ihara K, Nonaka K, et al. Therapeutic potential of hepatocyte-like-cells converted from stem cells from human exfoliated deciduous teeth in fulminant Wilson’s disease. Sci Rep. 2019;9:1535.

4. Iwanaka T, Yamaza T, Sonoda S, Yoshimaru K, Matsuura T, Yamaza H, et al. A model study for the manufacture and validation of clinical-grade deciduous dental pulp stem cells for chronic liver fibrosis treatment. Stem Cell Res Ther. 2020;11:134.

5. Miura M, Gronthos S, Zhao M, Lu B, Fisher LW, Robey PG, et al. SHED: stem cells from human exfoliated deciduous teeth. Proc Natl Acad Sci U S A. 2003;100:5807–12.

6. Choi KM, Seo YK, Yoon HH, Song KY, Kwon SY, Lee HS, et al. Effect of ascorbic acid on bone marrow-derived mesenchymal stem cell proliferation and differentiation. J Biosci Bioeng. 2008;105:586–94.

7. Yamaza T, Kentaro A, Chen C, Liu Y, Shi Y, Gronthos S, et al. Immunomodulatory properties of stem cells from human exfoliated deciduous teeth. Stem Cell Res Ther. 2010;1:5.

8. Dominici M, Le Blanc K, Mueller I, Slaper-Cortenbach I, Marini FCF, Krause DS, et al. Minimal criteria for defining multipotent mesenchymal stromal cells. The International Society for Cellular Therapy position statement. Cytotherapy. 2006;8:315–7.

9. Yamaza T, Ren G, Akiyama K, Chen C, Shi Y, Shi S. Mouse mandible contains distinctive mesenchymal stem cells. J Dent Res. 2011;90:317–24.

| Antibody names, antigen | Antibody types, host, clone name | Supplier names |
| --- | --- | --- |
| ACTB, human  BGLAP, human  Calnexin antibody, mouse | IgG2a, mouse, AC-74  IgG, polyclonal  IgG1, rabbit, EPR21205 | Millipore Sigma (St Louis, MO)  Abcam (Cambridge, UK)  Abcam (Cambridge, UK) |
| CD14 antibody, mouse | R-PE-conjugated IgG2a kappa, rat, M14-23 | Biolegend (San Diego, CA) |
| CD34 antibody, mouse | R-PE-conjugated IgG2a kappa, rat, MEC14.7 | Biolegend (San Diego, CA) |
| CD45 antibody, mouse | R-PE-conjugated IgG2b kappa, rat, 30-F11 | Biolegend (San Diego, CA) |
| CD63 antibody, rabbit | IgG, polyclonal | System Bioscience (Palo Alto, CA) |
| CD81 antibody, rabbit | IgG, polyclonal | System Bioscience (Palo Alto, CA) |
| CD73 antibody, mouse | R-PE-conjugated IgG1 kappa, rat, TY/11.8 | Biolegend (San Diego, CA) |
| CD90 antibody, mouse | R-PE-conjugated Ig G2b kappa, rat, 30-H12 | Biolegend (San Diego, CA) |
| CD105 antibody, mouse | R-PE-conjugated IgG2a kappa, rat, MJ7/18 | Biolegend (San Diego, CA) |
| CD146 antibody, mouse | R-PE-conjugated IgG2a kappa, rat, ME-9F1 | Biolegend (San Diego, CA) |
| CD146 antibody, human | R-PE-conjugated IgG1 kappa, mouse, P1H112 | Biolegend (San Diego, CA) |
| Histone 3, human  RUNX2 antibody, human  TERT antibody, human  IgG1 kappa, mouse | IgG, polyclonal  IgG, polyclonal  IgG, rabbit, Y182  R-PE-conjugated IgG1 kappa, mouse, MOPC-21 | Abcam (Cambridge, UK)  Abcam (Cambridge, UK)  Abcam (Cambridge, UK)  Biolegend (San Diego, CA) |
| IgG1 kappa, rat | R-PE-conjugated IgG1 kappa, mouse, MRG1-58 | Biolegend (San Diego, CA) |
| IgG2a kappa, rat | R-PE-conjugated IgG1 kappa, mouse, RTK2758 | Biolegend (San Diego, CA) |
| IgG2b kappa, rat | R-PE-conjugated IgG1 kappa, mouse, RTK4530 | Biolegend (San Diego, CA) |
| ACTB: actin, β; BGLAP, bone gamma carboxyglutamic acid protein; RUNX2: runt-related transcription factor 2;  R-PE: R-phycoerythrin; TERT, telomerase reverse transcriptase | | |

**Supplementary Table 1.** Specific antibodies for flow cytometry and Western blotting

**Supplementary Table 2.** TaqMan probes used for the mouse genes

| **Gene names** | **Gene assay ID Numbers** |
| --- | --- |
| *Bglap* | Mm03413826_mH |
| *Ctsk* | Mm000484039_m1 |
| *Nftac1* | Mm01265944_m1 |
| *Rank* | Mm00437132_m1 |
| *Runx2* | Mm00501584_m1 |
| *Sema3a* | Mm00436469_m1 |
| *Tert* | Mm00436931_m1 |
| 18S ribosomal RNA | Mm03928990_g1 |

Bglap, bone gamma carboxyglutamate protein; Ctsk, cathepsin k, Nftac1, nuclear factor of activated T cells 1; Rank, receptor activator of nuclear factor kappa; Runx2, runt related transcription factor 2; Sema3a, Semaphorin-3a; Tert, telomerase reverse transcriptase.

**Supplementary Table 3.** TaqMan probes used for the human genes

| **Gene names** | **Gene assay ID Numbers** |
| --- | --- |
| *BGLAP* | Hs00609452_g1 |
| *RUNX2* | Hs01047973_m1 |
| *SEMA3A* | Hs00173810_m1 |
| *TERT* | Hs00972056_m1 |
| 18S ribosomal RNA | Hs03003631_g1 |

BGLAP, bone gamma carboxyglutamate protein; RUNX2, runt related transcription factor 2; SEMA3a, Semaphorin-3a; TERT, telomerase reverse transcriptase.
